# Self-Expandable Metallic Stent Implantation Combined With Bronchial Artery Infusion Chemoembolization in the Treatment of Lung Cancer With Complete Atelectasis

**DOI:** 10.3389/fonc.2021.733510

**Published:** 2022-01-12

**Authors:** Xiaobing Li, Meipan Yin, Pengfei Xie, Ying Liu, Xiangnan Li, Yu Qi, Yaozhen Ma, Chunxia Li, Gang Wu

**Affiliations:** ^1^Department of Interventional Radiology, The First Affiliated Hospital of Zhengzhou University, Zhengzhou, China; ^2^Department of Respiratory, The First Affiliated Hospital of Zhengzhou University, Zhengzhou, China; ^3^ Department of Thoracic Surgery, The First Affiliated Hospital of Zhengzhou University, Zhengzhou, China

**Keywords:** lung cancer, atelectasis, self-expandable metallic stent, bronchial artery infusion chemoembolization, interventional radiology

## Abstract

**Background:**

Atelectasis is a common complication of lung cancer, and there are few reports about the treatment methods. This study retrospectively analyzed the safety and effectiveness of endotracheal metal stent implantation combined with arterial infusion chemoembolization in the treatment of non-small cell lung cancer with complete atelectasis.

**Methods:**

The clinical data of patients with non-small cell lung cancer and complete atelectasis treated by self-expandable metallic stent implantation combined with arterial infusion chemotherapy were retrospectively analyzed. The clinical efficacy was evaluated and postoperative adverse reactions were observed. Progression-free survival and overall survival were analyzed by Kaplan-Meier method.

**Results:**

In all, 42 endotracheal metallic stents were implanted in 42 patients under fluoroscopy. 5–7 days after stent implantation, CT showed that 24 patients (57.1%) had complete lung recruitment, and that 13 (31.0%) had partial lung recruitment. The technical success rate was 100%, and the clinical success rate was 88.1% (37/42). 5–7 days after stent implantation, bronchial artery infusion chemoembolization was performed in all patients. The median progression-free survival and overall survival were 6 months (95% CI: 2.04-9.66) and 10 months (95% CI: 7.22-12.79), respectively.

**Conclusion:**

Self-expandable metallic stent implantation combined with arterial infusion chemoembolization may be an effective and safe strategy in the treatment of lung cancer with atelectasis clinically.

## Introduction

Atelectasis in lung cancer often results from severe tracheal or bronchial obstruction due to cancer invasion. Subsequently, diminished alveolar air severely leads to substantial lung tissue damage, such as atrophy and collapse. According to associated reports, the incidence rate of lung cancer is 10–40% (1–3). The survival time of untreated lung cancer patients with atelectasis isn’t beyond 2 months, and patients often die of asphyxia, infection, or ventilator support-related complications (4). Lung cancer with complete atelectasis refers to the atelectasis of the whole lung, which often results in a dyspneic symptom. As the common therapeutic methods, radiotherapy or intravenous chemotherapy can hardly relieve airway obstruction in a short time, in contrast with the potential local tissue swelling and aggravate dyspnea subsequently.

Self-expandable metallic stent (SEMS) is widely used in the treatment of airway stenosis due to its significant advantage in the relieving airway stenosis and dyspnea (5–9). However, there are few reports on SEMS being applied in the treatment of lung cancer with atelectasis (10), because the SEMS cannot suppress the invasion of tumor tissue to the surrounding areas. The spread of cancer through the mesh of the stent causes airway restenosis, which affects the long-term efficacy of SEMS in the treatment of atelectasis.

Bronchial arterial transcatheter arterial chemoembolization (BA-TACE) infuses chemotherapeutic drugs directly into the tumor-feeding artery to increase the local drug concentration and destroy cancer cells effectively. It has been demonstrated that infusion chemotherapeutic drugs in small doses ensures the therapeutic effect and avoid severe side effects. Bronchial artery embolization can further improve the curative effect by blocking the tumor-feeding artery and tumor vascular bed (11–13). This study evaluated the safety and efficacy of SEMS implantation combined with BA-TACE in the treatment of lung cancer with complete atelectasis, which provides a preliminary clinically evidence for promotion the combined therapy.

## Materials and Methods

### Patients

In this retrospective study, we analyzed the clinical data of patients with lung cancer with complete atelectasis who received the combined therapy in the Department of Interventional Radiology of our hospital, from June 2012 to May 2020. Patients’ medical records, imaging data, operation records, and follow-up results were analyzed. The following inclusion criteria were applied: ① non-small cell lung cancer diagnosed by histological examination; ② complete atelectasis confirmed by CT imaging; and ③ sequential treatment combined with SEMS implantation and BA-TACE. The exclusion criteria were as follows: ① patients without BA-TACE after stent implantation; ② patients without stent implantation before BA-TACE; and ③ patients who received any other type of treatments in the course of the combined therapy.

This study protocol was approved by the ethics investigation committee of the First Affiliated Hospital of Zhengzhou University. Ethical approval code: SS-2018-25. Written informed consent was obtained from each patient during questionnaire administration for the collection and analysis of applicable clinical data.

### Preoperative Preparation

Blood routine, electrolytes, coagulation function, tumor markers, liver and kidney function, and electrocardiogram results were examined before the operation. The location, degree, and length of stenosis, atelectasis, and pleural effusion were confirmed by plain and enhanced chest CT. In this study, all stents used were bare. The diameter of the stent was about 10% larger than that of the trachea and main bronchus in the mediastinal window of chest CT. The airway obstruction is at least 1 cm away from the carina or larynx, a straight tubular tracheal stent should be used; the obstruction in the carina area, a Y-shaped endotracheal stent is preferred, and the obstruction in distal of the main bronchus, a small Y-shaped tracheal stent should be used. The SEMS used in the study was manufactured by Nanjing Micro-Tech Medical Company (Nanjing City, Jiangsu Province, China).

### SEMS Implantation Therapy

The patients were administered intramuscular diazepam (10 mg) and anisodamine (10 mg) and intravenous dexamethasone (10 mg), 30 min before the procedure. The procedures were performed under fluoroscopic guidance, without the use of bronchoscopy. Interventional radiologists placed the stent under local anesthesia. Patients lay on the examination bed, with ECG monitoring. A gag was used to open the mouth. Oxygen was administered *via* a nasal catheter, and a sputum aspirator was prepared. Under fluoroscopy, a 0.035-inch hydrophilic guide wire (Cook Corporation, Bloomington, IN, USA) and a 5 F vertebral artery catheter (Cordis Company, New Jersey, USA) were introduced transorally into the trachea or bronchus. Then, 5 mL of 2% lidocaine and 5 mL of 0.01% epinephrine were quickly sprayed *via* the catheter. Tracheography was performed to determine the location of airway stenosis. After the guide wire and catheter pass through the stenosis, the guide wire was withdrawn. Retract the catheter while injecting contrast agent for airway imaging. We could use this technique to define the distal end of the stenotic component of the main bronchus. Exchange with stiff guide wire into one side bronchus, 9 F sheath tubes was placed along guide wire. Another 0.035-inch hydrophilic hard wire was introduced for Y-shaped or small Y-shaped stenting. In general, the bronchial component of the stent was slowly released 0.5 to 1 cm beyond the distal end of the stenotic component of the main bronchus. After the stent was successfully inserted, the sputum aspiration tube was introduced for sputum drainage to avoid suffocation (5, 14).

The patients were given aerosol inhalation and anti-inflammatory treatment after SEMS implantation, and their vital signs were closely monitored. A repeat CT was performed 5–7 days after the implantation to observe the position of the stent, degree of expansion, and lung recruitment.

### Bronchial Artery Infusion Chemoembolization

The BA-TACE procedure was performed 5–7 days after SEMS implantation and when the dyspnea was relieved. BA-TACE chemotherapy regimen: comprised epirubicin 30–50 mg, nedaplatin 40–60 mg, and raltitrexed 4 mg.

The Seldinger technique was used to puncture the femoral artery. The location of the tumor- feeding artery was determined by bronchial arteriography. If necessary, a 2.7-F micro guide superselective catheter was used. The dose of chemotherapy drugs is decided during the procedure on the basis of the number of blood supply arteries and the degree of tumor staining (15). Each drug was dissolved in 150–200 mL solution and injected into the tumor-feeding artery at a constant rate of 10 mL/min. Subsequently, 350–560 µm gelatin sponge particles (in absence of hemoptysis) or PVA particles (in presence of hemoptysis) were used to embolize the tumor-feeding artery (13).

The patients were treated with proton pump inhibitors, antiemetics, antibiotics, and expectorants. BA-TACE was performed 1–3 times at 4-week intervals according to the degree of tumor shrinkage. Chest CT was reexamined before each BA-TACE to evaluate atelectasis and its curative effect on the tumor.

### Evaluation

The chest CT and DSA images were analyzed by two experienced interventional radiologists. The curative effect of lung recruitment was divided into complete lung recruitment, partial lung recruitment, no lung recruitment, and progressive atelectasis (16). Dyspnea patients were divided into five grades based on dyspnea score (17). In this study, progression-free survival (PFS) and overall survival (OS) were used as the outcome measures. After treatment with airway stenting and BA-TACE, absence of any serious operation-related event was considered as technical success. An improvement of clinical symptoms or of the atelectasis on CT after SEMS implantation was considered as clinical success.

The adverse events during and after treatment were recorded in detail. Adverse events were graded according to the American standard for common adverse reaction terminology (version 5.0).

### Statistical Analysis

SPSS software (version 23.0, IBM, Armonk, NY, USA) was used for all statistical analysis. Data are presented as medians, mean ± standard deviation, or percentages. Overall survival (OS) was calculated by the Kaplan-Meier method. OS was calculated from the day of histologic diagnosis to the date of death or last follow-up. P values <0.05 indicated statistical significance.

## Results

A total of 42 patients [33 males and 9 females, age: 37–86 (mean: 60.6 ± 11.32) years] were enrolled in this study. There were 30 cases of dyspnea (dyspnea score > 1), 15 cases of cough (35.7%), four cases of hemoptysis (9.5%), two cases of eating obstruction (4.8%), one case of chest pain (2.4%), and one case of fever (2.4%). Among those with comorbid diseases, seven patients had hypertension (16.7%), eight had type 2 diabetes (19.0%), one had coronary heart disease (2.4%), and three had chronic lung disease (7.1%).

The clinical characteristics of the patients are shown in **Table 1**.

**Table 1:** Baseline Characteristics of Study Patients (N=42).

Variables	Data
Histological type	
Squamous cell carcinoma	33 (78.6)
Adenocarcinoma	8 (19.0)
Adenosquamous carcinoma	1 (2.4)
TNM stage	
III	24 (57.1)
IV	28 (66.7)
Dyspnea classification(N=30)	
2	4 (9.3)
3	17 (39.5)
4	6 (14.0)
5	3 (7.0)
Location of atelectasis	
Right lung	28 (65.1)
Left lung	14 (33.3)
Previous treatment	
Chemotherapy	22 (52.4)
Radiotherapy	5 (11.6)
Surgery	5 (11.6)
^125^I seed implantation	4 (9.3)

### SEMS Implantation and Clinical Results

In all, 42 SEMS were implanted in 42 patients under fluoroscopic guidance, including four straight tubular stents, five L-shaped stents, 31 large Y-shaped stents, and two small Y-shaped stents. The success rate of stent implantation was 100%. The operation time of stent implantation ranged from 4–42 (mean: 14.2 ± 7.11) minutes. There were no serious events such as massive hemorrhage, asphyxia, or death related to the operation. The status of stent placement, stent type, location, and degree of airway stenosis are shown in **Table 2**. The types and sizes of stents are shown in **Table 3**.

**Table 2:** Statistics of stent placement.

Data	
Emergency	10 (23.8)
Non-emergency	32 (76.2)
Severity of airway obstruction	
III	10 (23.8)
IIV	12 (28.6)
Narrow part	
Trachea + Carina	9 (21.4)
Carina + Right main bronchus	5 (11.9)
Carina + Light main bronchus	9 (21.4)
Carina + Left and right bronchus	10 (23.8)
Light main bronchus	3 (7.1)
Right main bronchus	6 (14.3)

**Table 3:** The stent types and dimensions.

Stent Types	n	Median Diameter (mm)	Median Length (mm)
Straight tubular stents	4	20 (20-20)	50 (40-60)
L-shaped stents	5	Main tube 20 (20-20) Branches 13 (10-14)	Main tube 40 (30-60) Branches 25 (20-40)
Large Y-shaped stents	31	Main tube 22 (12-22) Left branches 12 (8–14) Right branches 12 (10–14)	Main tube 40 (30-55) Left branches B30 (10–35) Right branches 15 (10–50)
Small Y-shaped stents	2	Main tube 12 (12-12) Branches 10 (10-10)	Main tube 22.5 (20–25) Branches 13 (10–15)

After 5 to 7 days of stent implantation, CT showed that 24 patients (57.1%) had complete lung recruitment, and 13 patients (31.0%) had partial lung recruitment. The technical success rate was 100%, and the clinical success rate was 88.1% (37/42). Five patients (11.9%) had no lung recruitment; hence, they were treated with sputum aspiration and ablation under fiberoptic bronchoscopy. Eventually, their clinical symptoms and atelectasis improved. The oxygen saturation was >94%, and the dyspnea score showed significant improvement, with 13, 11, and 6 patients showing a score of 0, 1, and 2.

### BA-TACE

Twenty-seven, 11, and 4 patients underwent one, two, and three BA-TACE procedure, respectively. A total of 70 arteries in 42 patients were confirmed as tumor-feeding arteries by angiography, with an average of 1.67 ± 0.55 arteries per patient (range: 1-3), including 46 bronchial arteries, 7 internal thoracic arteries, 1 esophageal artery, 1 thyroid neck trunk, and 15 intercostal arteries.

**Figure 1:**
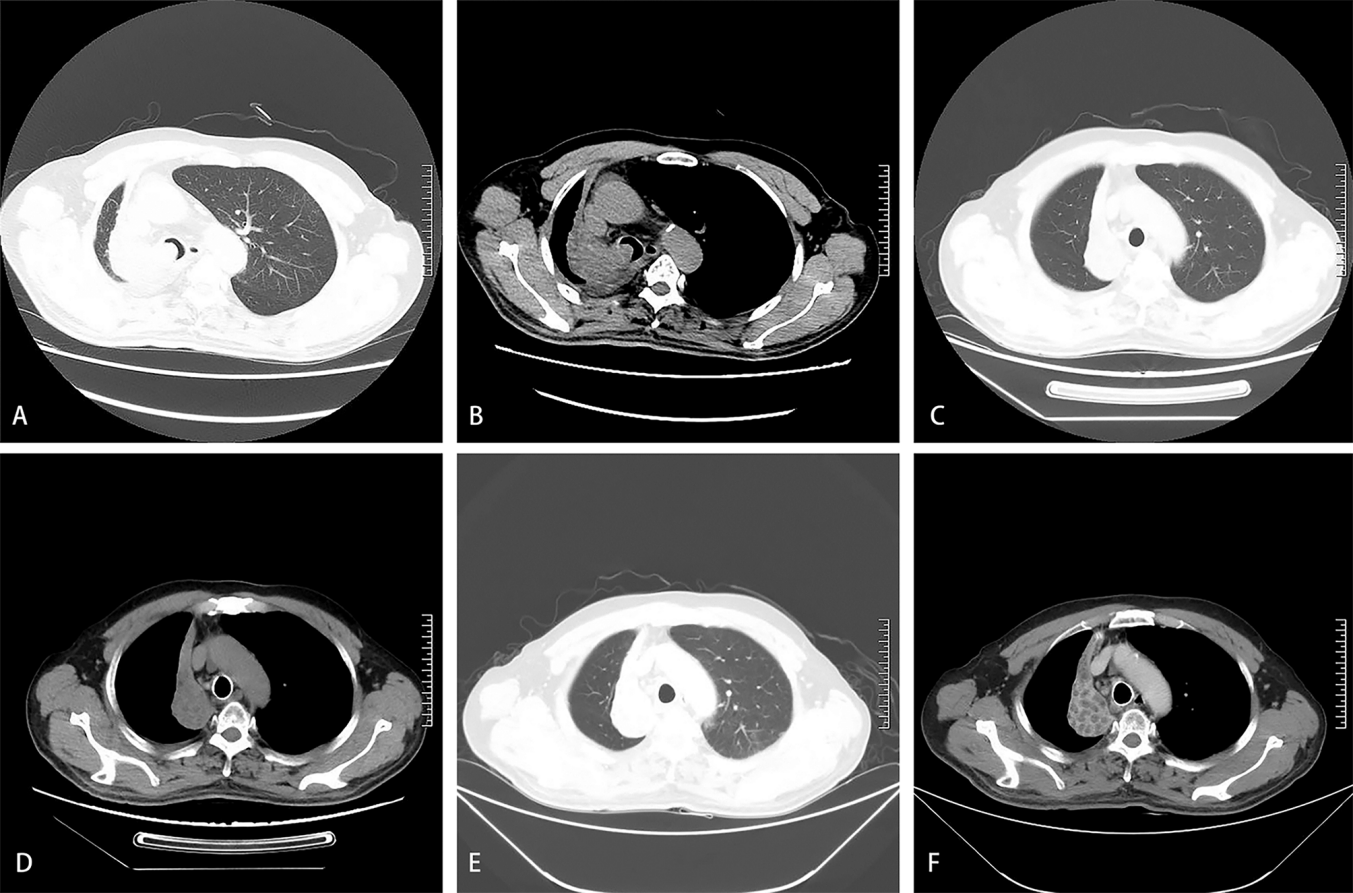
A 61-year-old man was diagnosed with squamous cell carcinoma of the right lung 8 months ago. He had progressive dyspnea for 3 days. The dyspnea score was 5. Chest computed tomography on admission showed complete atelectasis in the lung window **(A)** and the mediastinal window **(B)**. Repeated computed tomography in the lung window **(C)** and the mediastinal window **(D)** showed a reduction in the size of the tumor in the right lung one month after the first BA-TACE. Repeated computed tomography in the lung window **(E)** and the mediastinal window **(F)** showed obvious necrosis in the tumor area one month after the second BA-TACE.

**Figure 2:**
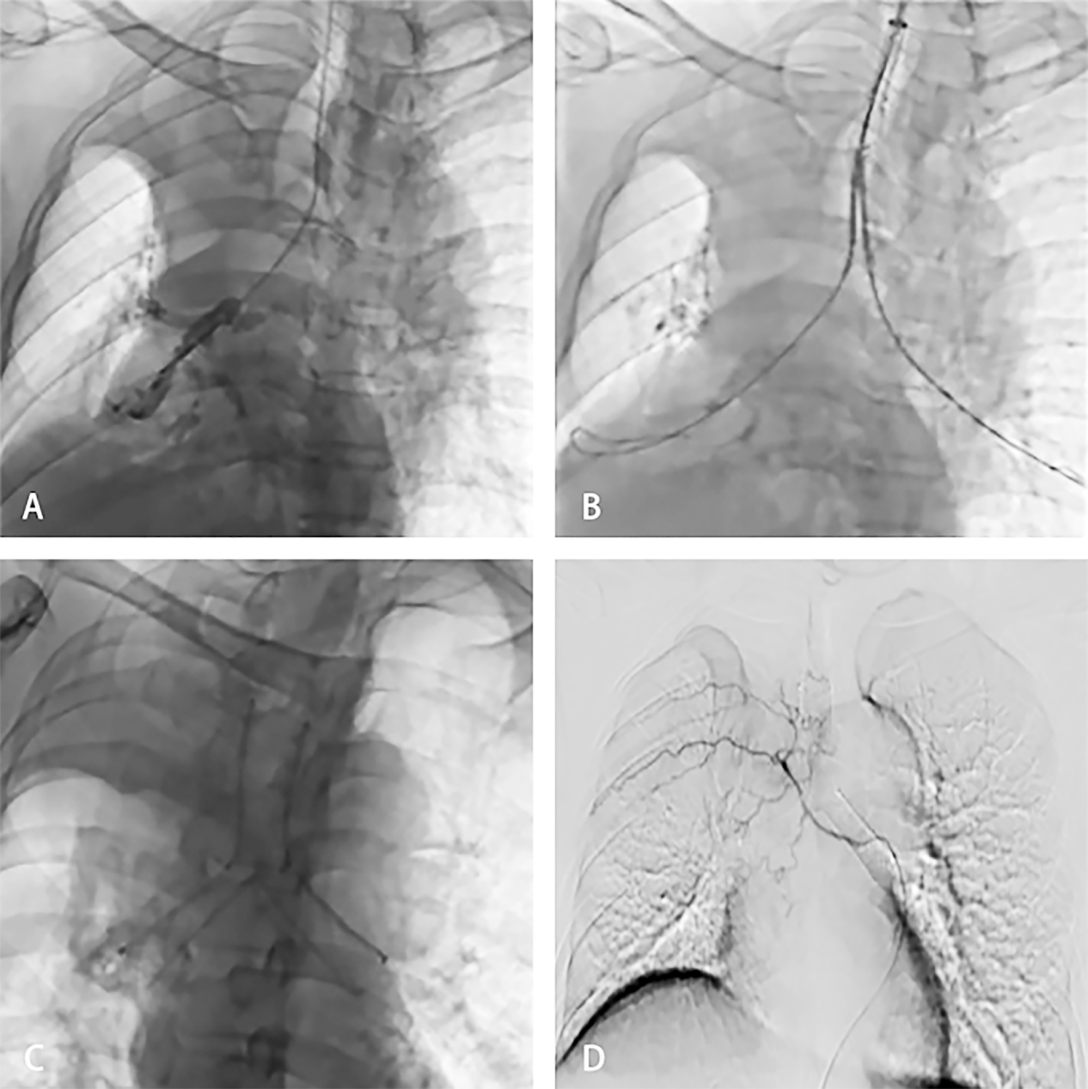
The patient was then treated with SEMS implantation and BA-TACE. Bronchography showed complete blockage of the distal right main bronchus and carina **(A)**. The stent delivery system was inserted under fluoroscopic guidance to reach the right blocked bronchus **(B)**. Fluoroscopy showed release of the Y-shaped stent **(C)**. Arteriography showed that the arteries were thickened and areas of abnormal staining were visible **(D)**.

**Figure 3:**
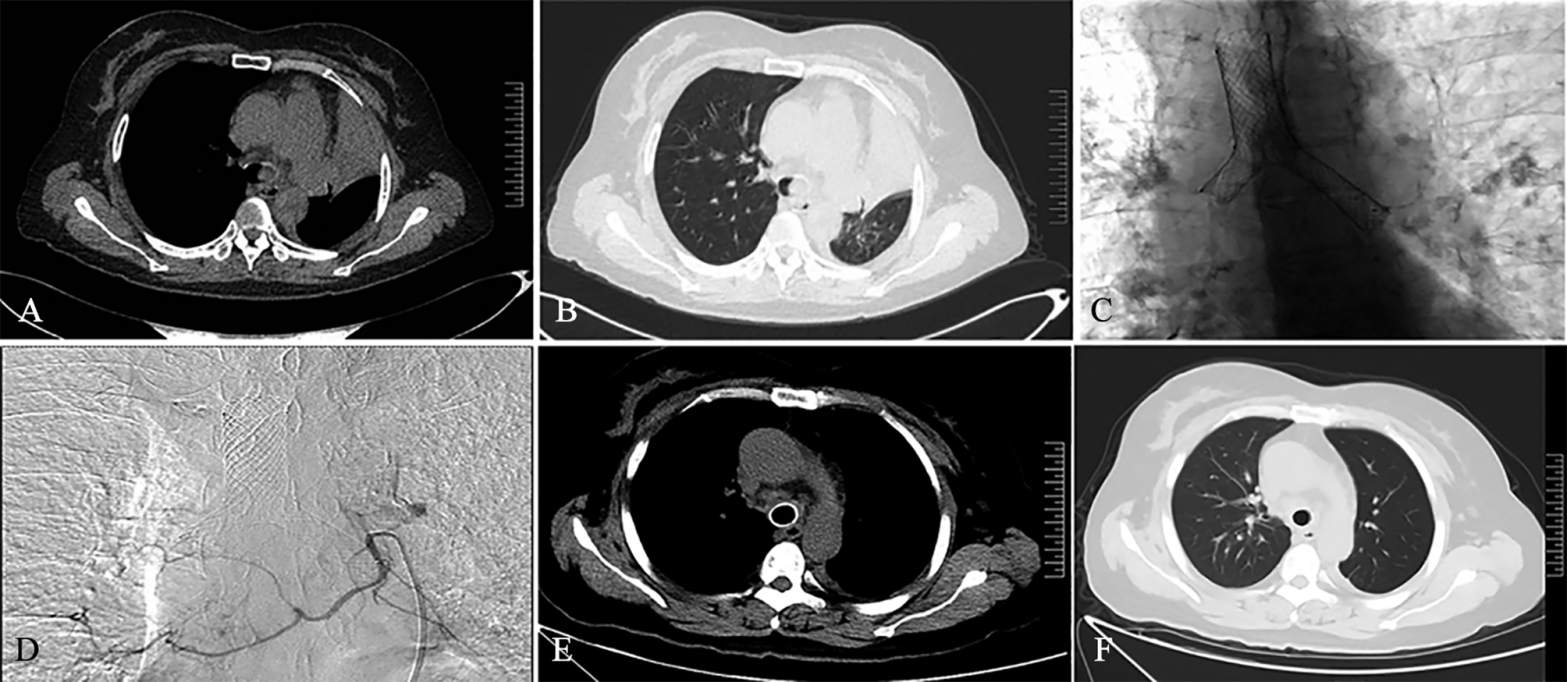
A 45-year-old female patient was diagnosed with left lung squamous cell carcinoma 2 weeks ago. She had dyspnea for 1 week. Chest computed tomography on admission showed atelectasis on the left side in the mediastinal window **(A)** and lung window **(B)**. The patient subsequently received SEMS implantation **(C)** and BA-TACE **(D)** treatment. One month after treatment, the patient’s reexamination of CT showed complete left lung recruitment in the mediastinal window **(E)** and the lung window **(F)**, and the tumor treatment effect was complete remission.

All 42 patients were successfully treated with chemotherapy *via* the tumor-feeding artery: 38 patients were treated with gelatin sponge and four were treated with PVA particles. Typical cases are shown in **Figures 1**–**3**.

Four weeks after the last BA-TACE treatment, the patient received subsequent treatment. Eight patients received targeted therapy, 10 received intravenous chemotherapy, eight received radiotherapy, five received radioactive seed implantation, six received chemotherapy and PD-1 treatment, and six did not receive any anti-tumor treatment until the end of follow-up.

### Survival

**Figure 4:**
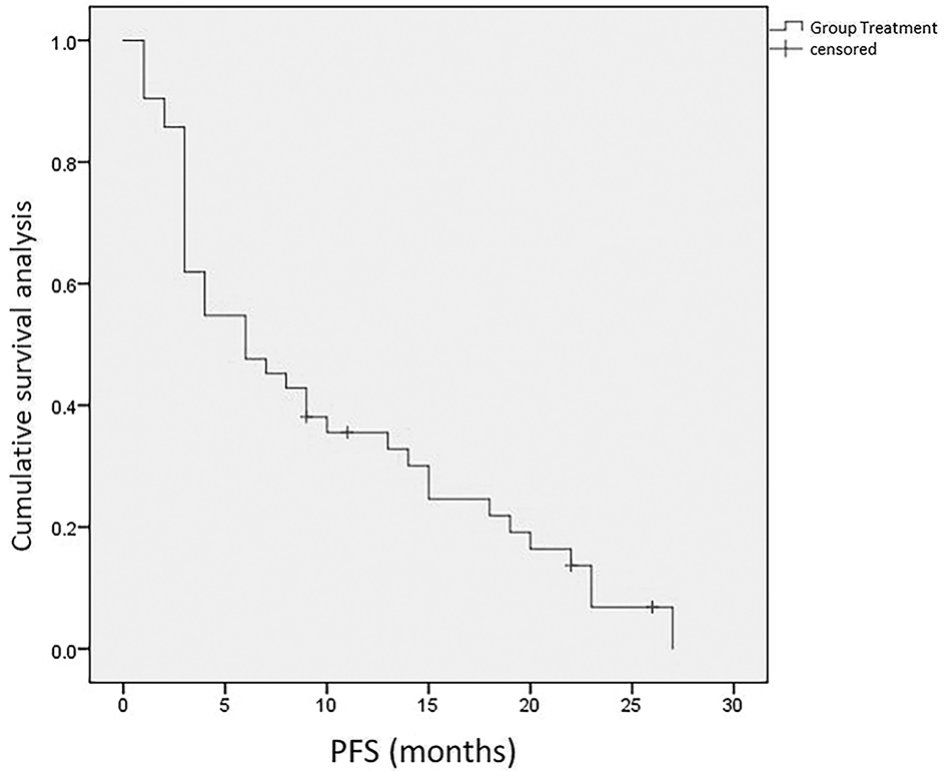
Kaplan-Meier curve for PFS.

**Figure 5:**
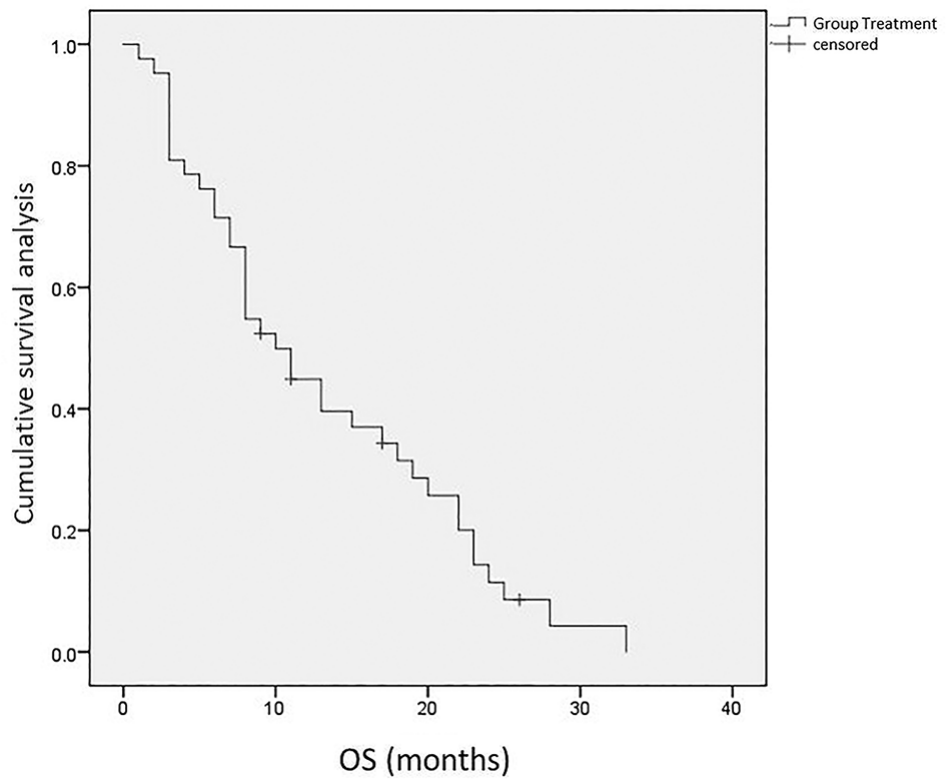
Kaplan-Meier curve for OS.

The median PFS was 6.0 months (95% CI: 2.04–9.66), and the median OS was 10.0 months (95% CI: 7.22–12.79). The 6- and 12-month survival rates were 71.4% and 42.3%, respectively (**Figures 4**, **5**).

Until the end of the follow-up period, four patients survived and were followed up for 9–26 months. Dyspnea did not recur in these 4 patients. In all, 38 patients died of the following causes: massive hemoptysis (n=1), cerebrovascular accident (n=3), pulmonary infection (n=14), cardiac arrest (n=4, one had coronary heart disease), heart failure (n=3), and cachexia caused by tumor (n=13). Among the 39 patients who died, 18 experienced recurrent dyspnea.

### Adverse Events

Eight patients (19.0%) had poor expectoration after SEMS implantation, which improved after sputum suction under bronchoscopy. The postoperative adverse events included hemoptysis, nausea, vomiting, fever, elevated serum alanine, and aspartate aminotransferase levels, and decreased platelet count. All the adverse events were classified as grade 1 (**Table 4**).

**Table 4:** Adverse events and subsequent treatment (N=42).

Adverse events	Data	Treatment	Effect
Intraoperative			
cough	11(26.2)	10 mg dexamethasone intravenous bolus	relief
Postoperative			
Grade 1 nausea/vomiting	9 (21.4)	Antiemetic treatment	relief
Grade 1 fever	6 (14.3)	Antipyretic treatment	relief
ALT/AST increase			
Grade 1	5 (11.9)	Hepatoprotective treatment	relief
Thrombocytopenia Grade 1	3 (7.1)	Platelet Ascending treatment	relief
Chest pain grade 1	13 (31.0)	Symptomatic treatment	relief
Abdominal pain grade 1	1 (2.4)	Symptomatic treatment	relief
Hemoptysis	6 (14.3)	Hemostatic treatment	relief

## Discussion

In lung cancer progression, atelectasis with dyspnea and insufficient ventilation is a common complication, affecting the quality of life (18). Owing to complication, old age, physical status, and other factors, traditional radiotherapy and chemotherapy are not effective for lung cancer with atelectasis (19). Previous report has exhibited the low efficiency of molecular targeted therapy (e.g., tyrosine kinase inhibitors) (20). The efficacy of other treatment methods such as radioactive seed implantation between tumor tissues and traditional Chinese herb in the treatment of advanced lung cancer remains to be verified (21–23).

Fast and accurate placement of the intraairway stent guarantees the successful operation. Thus, the successful placement of stent under fluoroscopy requires highly skilled operators. As a palliative interventional therapy, stent placement can rapidly relieve atelectasis caused by airway obstruction and improve ventilation, but there are related complications (24), such as irritative cough, stimulating granulation tissue hyperplasia, tracheal perforation, hemoptysis, asphyxia, and death, which ultimately influence the long-term curative effect. The stent implantation can only improve dyspnea and provide opportunities for later treatment.

Pulmonary tumors are considered to be fed through two different vascular systems, comprising low-pressure pulmonary arterial circulation and high-pressure systemic arterial circulation, including the bronchial artery (1, 25). BA-TACE is considered as a good choice for the treatment of advanced lung cancer. BA-TACE has a high rate of successful tumor reduction in a short time, less adverse reactions, and high repeatability (13, 26, 27). BA-TACE combined with SEMS can not only suppress tumor but also relieve symptoms, improve lung recruitment rate, and maintain the airway stent patency rate. Arteriography to find all the tumor-feeding arteries plays a key role in BA-TACE treatment. We judge whether all the arteries have been found based on the consistency of the position and shape of the tumor presented in the arteriography and CT.

**Figure 6:**
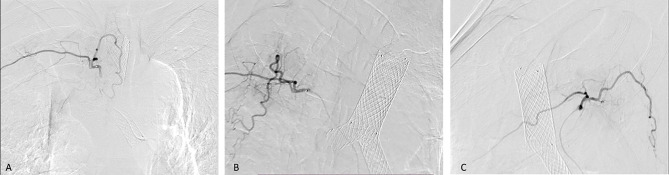
Bilateral bronchial arteriography shows the vascular distribution of the embolized tumor. A 57-year-old man was diagnosed with right lung squamous cell carcinoma and right atelectasis. He underwent BA-TACE after airway stent placement. In Figures **(A–C)**, we use bilateral bronchial angiography to find the vascular distribution of the tumor, and use a microcatheter for infusion chemoembolization.

However, BA-TACE may lead to serious complications such as cerebral infarction, spinal cord injury, esophageal perforation, and tracheal perforation (28, 29). Identifying the spinal artery and the abnormal communication between the bronchial artery and the pulmonary vein is a prerequisite to avoid serious adverse events such as paraplegia and cerebral infarction. According to our experience, careful operation, use of microcatheter when necessary (Typical cases are shown in **Figures 6**), and selection of types and models of embolic materials (the selection of permanent embolic materials for patients with hemoptysis, and the use of gelatin sponge for patients without hemoptysis) are details that result in a high technical success rate and low incidence of adverse events (30).

There are few reports on the clinical efficacy of sequential treatment of lung cancer with atelectasis by SEMS implantation and BA-TACE. There is only one report about retrospective analysis of bronchoscopic implantation of ^125^I radioactive seeds for the treatment of lung cancer with complete atelectasis (16), the limitation of this technique is that it can only be applied to patients with mild dyspnea and without hemoptysis.

The main limitation of this study is that it is a single center, retrospective, and observational analysis, with a limited number of cases and no control group for comparison. More prospective and large-scale clinical controlled trials are needed in the future.

This study shows that SEMS combined with BA-TACE is an effective and safe method in the treatment of non-small cell lung cancer with atelectasis, which can be attempted clinically. Large-scale randomized clinical trials need to be carried out to further confirm the results of this study.

## Data Availability Statement

The original contributions presented in the study are included in the article/supplementary material. Further inquiries can be directed to the corresponding author.

## Ethics Statement

This study protocol was approved by the ethics investigation committee of the First Affiliated Hospital of Zhengzhou University. Ethical approval code:SS-2018-25. The patients/participants provided their written informed consent to participate in this study.

## Author Contributions

XiaoL and GW contributed in the study design, data collection, data analysis, data interpretation, literature search, and writing of the article. MY contributed in the literature search and writing of the article. PX and YL contributed in the data collection, analysis, and interpretation. XiangL, YQ, YM, and CL contributed in the data analysis and data interpretation. All authors contributed to the article and approved the submitted version.

## Conflict of Interest

The authors declare that the research was conducted in the absence of any commercial or financial relationships that could be construed as a potential conflict of interest.

## Publisher’s Note

All claims expressed in this article are solely those of the authors and do not necessarily represent those of their affiliated organizations, or those of the publisher, the editors and the reviewers. Any product that may be evaluated in this article, or claim that may be made by its manufacturer, is not guaranteed or endorsed by the publisher.
